# Zhu Xi: a pioneer of experimental biology

**DOI:** 10.1093/procel/pwac055

**Published:** 2022-12-31

**Authors:** Yujing Qian, Xiazhao Yu, Yangqing Sun, Qun Xie, Ge Liang, Gaiping Bai

**Affiliations:** Teacher Education College, Zhejiang Normal University, Jinhua 321000, China; Teacher Education College, Zhejiang Normal University, Jinhua 321000, China; Teacher Education College, Zhejiang Normal University, Jinhua 321000, China; Teacher Education College, Zhejiang Normal University, Jinhua 321000, China; Teacher Education College, Zhejiang Normal University, Jinhua 321000, China; Teacher Education College, Zhejiang Normal University, Jinhua 321000, China

Zhu Xi (朱洗, 1900–1962) (Fig. 1), one of the founders of cell biology and experimental biology in China, was born in Linhai, Zhejiang Province in 1900. In 1931, he obtained his doctorate in France. After returning China, he devoted himself to toad reproduction research. With the spirit of innovation and dedication to research, he had made marvelous contributions to China, especially in biology. At the same time, he put his patriotism into action, promoting the vigorous development of agriculture and other industries.

**Figure 1.**
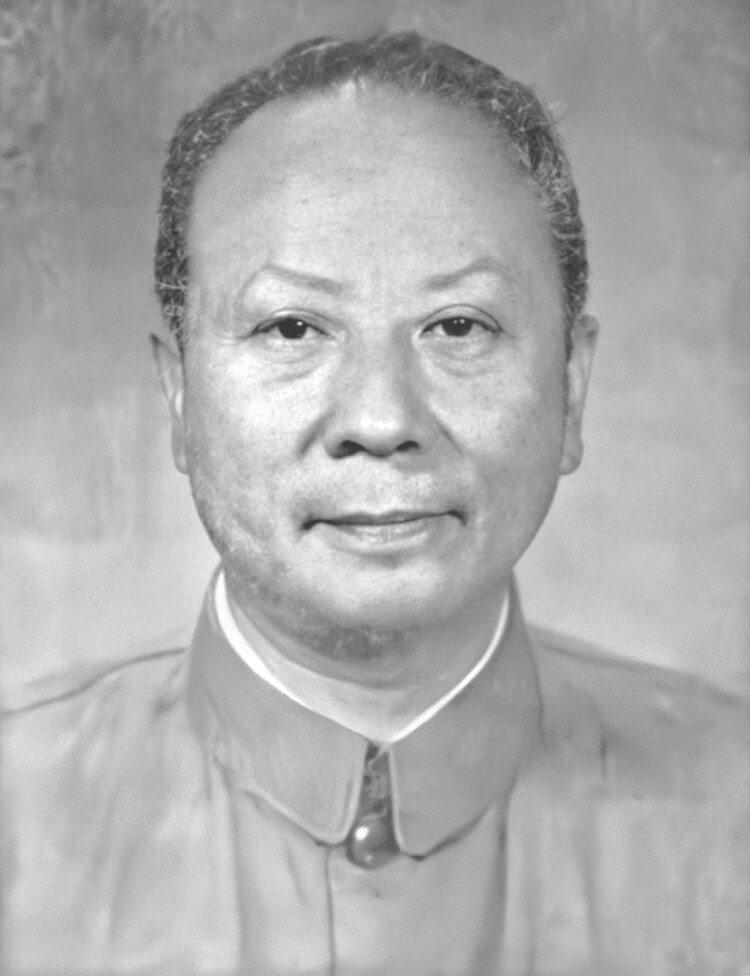
Zhu Xi (1900–1962), from Tong (1962).

## The life of Zhu Xi

Zhu Xi was born on October 14, 1900 in Dianqian village of Linhai, Zhejiang. For those who enjoy learning, in fact, learning has become part of their meaningful lives. Zhu Xi is exactly that kind of person. In his early years, influenced by traditional Chinese culture, he studied at a private school and started to study Confucian classics. At the age of 15, he enrolled to the Hui Pu School, a local county school, owing to excellent grades. Three years later, he was admitted to Zhejiang No. 6 Middle School. In 1919, the May 4th Movement took place in China. Zhu Xi joined the strike but was expelled from the school. However, his passion for learning never faded away no matter what he suffered. At the time, Zhu Xi, as a typesetter in Linhai, responded to call of Cai Yuanpei (蔡元培), Li Shizeng (李石曾) and others and rushed to Shanghai to apply for going to Europe without hesitation. In 1920, Zhu Xi was sent to study abroad by virtue of his great intelligence and capacity.

All things are difficult before they are easy. After his arriving in France, financial hardship impelled him to make a living. He took many jobs, worked hard during the day, and studied French and biology at night. Five years later, he managed to save enough money to go to Montpellier University, where he met Professor Jean Eugène Bataillon, a famous embryologist. Before long, Zhu Xi displayed his talent for the first time in biology, publishing about 14 papers with Professor Bataillon. His perseverance, diligence, concentration enabled him to move forward firmly in adversity.

Undoubtedly, the advanced experimental facilities in France paved the way for his research, and he soon became famous as a biologist. The Mukden Incident shocked the world. Zhu Xi firmly believed that saving the country as important as science, and declined Professor Bataillon’s detainment. He spent years of savings on books and laboratory equipment, which he brought back to China.

After returning to China, Zhu Xi worked at Sun Yat-sen University in Guangzhou, Institute of Zoology, Peking Research Institute, and Institute franco-chinois. He also set up a Biological Research Institute in Shanghai. At that time, China was suffering from internal and external troubles. Zhu Xi not only faced difficulties such as the shortage of experimental materials, but also had to avoid the attack of artillery fire. After the fall of Shanghai, Zhu Xi returned to Linhai, where although he was temporarily unable to engage in biological research, he continued to devote himself to running schools for his hometown. After the founding of the People’s Republic of China, Zhu Xi was appointed as a researcher at the Institute of Experimental Biology, Chinese Academy of Sciences, which provided him a relatively stable research environment. Before that, he had to frequently travel to many places to do instruction and research.

Zhu Xi held that scientific work requires all one’s life, and the 8-hour working day is not enough. In addition to cultivating the world’s first “toads without grandparents”, solving the problems of rearing Eri silkworms and breeding artificially farmed fish, he had written 25 books and translated 4 international classics. Even though he was plagued by illness in his later years, he continued to write until the end (Yang, 1992). Zhu Xi had devoted his whole life to biology and homeland.

## Zhu Xi’s contributions to experimental biology

Zhu Xi had a deep connection with biology. He was close to biology from an early age, when his family kept a variety of animals. Before learning from Professor Bataillon, he already studied evolutionism and neo-Lamarckism. People usually talk about Zhu Xi’s research on parthenogenesis in frogs, which he completed with Professor Bataillon, but in fact his most famous contribution in biology is the research on parthenogenesis in toads.

### The breeding of parthenogenetic toad

In 1951, Zhu Xi, along with his assistant Wang Youlan (王幽兰) et al., carried out the difficult research. Previously, although some researchers had used toad eggs as artificial parthenogenetic materials, the effect was disappointing. Zhu Xi also experimented with acupuncture on the eggs of two kinds of toads from Guangdong and Shanghai, and got similar results. But Zhu Xi was not discouraged, instead focused on improving acupuncture methods. He found that the gelatin layer surrounding toad eggs bands were too thick to penetrate with the usual fine needle, and naked eggs without membrane were delicate and not resistant to acupuncture. After careful consideration, Zhu Xi finally chose to use naked eggs and improved the fine needles because of its many advantages, such as the possibility of no fertilization. Therefore, after the initial failure of needling 6,840 toad eggs with platinum needles, resulting in only 13 malformed gastrulas that failed to develop further, let alone hatched tadpoles, Zhu Xi switched to glass needles with a smaller diameter. The needle, with a tip diameter of 10–13 μm, did not work well at first as an experimental tool, but after several refinements, it worked well in experiments. The next year, Zhu Xi’s team punctured 3,650 naked eggs with glass needles, and almost every time they obtained hatched tadpoles.

The period, 1958–1959, was actually the time when he started his formal work. During this period, Zhu Xi’s team mainly explored three questions: the performance of ovum activation, the acquisition and development of parthenogenetic toads, and the relationship between ovary maturation and parthenogenetic development. After puncturing 40,140 eggs, on the basis of careful observation and comparison, three difficult questions had been precisely solved. The most remarkable thing was that they managed to breed 25 parthenogenetic toads (Zhu and Wang, 1959). And the results suggested that toads can be used as research material in the same way that frogs had been used for parthenogenesis studies in the past.

Not only have a dream, but try to achieve it by working hard. In March 1961, the only remaining parthenogenetic toad, an adult female, laid more than 3,000 eggs, giving birth to the world’s first toads without a maternal grandfather (Zhu et al., 1961). Such satisfactory research results could be attributed not only to his innovation, but also to his dedication to research on the basis of predecessors.

### Introduction, domestication, and popularization of the Eri silkworm

Zhu Xi did not divorce himself from the masses and from reality and act blindly. On the contrary, he concerned about the real life and spared no effort to make contributions to improving people’s lives. He once called on his colleagues to study and breed Indian Eri silkworms according to the reality of China to promote agricultural economic development and improve the ecological environment. Then he took the lead in studying the methods of rearing and conservation of Eri silkworms, and put forward valid suggestions on the use of Eri silk (Zhu et al., 1953). Furthermore, he also crossed the Eri silkworm with native silkworms, and selected new silkworms of good traits.

Based on this theory and practice, he guided the silkworm farmers to raise Eri silkworms, composed brochures on its breeding, and set up training courses for its breeding in many cities and provinces. Finally, the introduction, domestication and popularization of Eri silkworm were successfully completed in the whole country.

### Studies on breeding of silkworms

Zhu Xi was devoting himself to the research and promotion of the Eri Silkworm, while still keeping a watchful eye on the native silkworm. In response to the call of “Science serves the people”, he led the research team to collect more than 50 kinds of plant leaves and raised silkworms one by one. In elaborate experiments, control experiments were conducted to compare the results of silkworm rearing of each plant leaves with that of mulberry leaves. In the case of the dandelion, he divided the silkworms which used in experiments into 6 groups. The 1st group and the 6th group ate dandelion and mulberry leaves respectively, the 2nd group ate mulberry leaves from its fifth instar, and the 3rd group ate mulberry leaves from its fourth instar, and so on (Zhu et al., 1951). The gradient control group experiment could not only compare the effects of two kinds of leaves on rearing silkworm, but also roughly establish the optimal rearing mode.

The same controlled experiment method was also used in the study of silkworm dieting disease. Zhu Xi noticed that the silkworm in Jiangsu and Zhejiang province often suffered from gastrointestinal diseases and the incidence was related to the season. To find an effective way to prevent the disease, he led his team designed and manufactured the “constant temperature and humidity chamber”, which allowed researchers to adjust the climate conditions by setting different temperatures and humidity. Then Zhu Xi divided silkworms which used in experiments into experimental groups and control groups, and the control group was given enough mulberry leaves, while the experimental group was given successively reduced mulberry leaves. Subsequently, he put these silkworms into the same chamber with high temperature and humidity, and the experimental results showed that silkworms were apt to disease when they were fed well in hot and humid conditions, and a proper diet could effectively avoid gastrointestinal diseases (Zhu et al., 1952). These comparative studies on the rearing of silkworms showed the wisdom and carefulness of this great pioneer of biology.

### Propagation of farmed fish

Even in peacetime, Zhu Xi’s research was still closely related to the economic development of the motherland and the livelihood of the people. His research on artificial reproduction of domestic fish also reflected his belief that research should be combined with practice. Zhu Xi and his research team visited nearby fish ponds and even distant rivers and lakes, and carried out a series of studies on the fertilization, maturation and embryonic development of goldfish, carp, and roach in order to solve the practical problem that the gonads of domestic fish were difficult to develop in ponds, and finally obtained satisfactory results (Zhu et al., 1960). In 1958, he used chorionic gonadotropin to induce the spawning of several kinds of domestic fish, which made silver carp and bighead carp spawn, and realized the artificial reproduction of domestic fish. Afterwards he established a whole set of artificial fish farming techniques and popularized them on a large scale, which brought the country’s freshwater farming industry to a new level.

Zhu Xi had been engaged in biological research for decades. During his lifetime, he wrote monographs like *Modern Biology Series*, translated famous works like *Embryology of Vertebrates*, and published popular science books like *Zoology*. He is an outstanding biologist whose spirit inspires generations of young people. He promoted the development of cell biology and experimental biology in China, and contributed to the popularization of science. Zhu Xi is also a patriot who has a deep affection for his country. No matter when and where, he closely combined his destiny with biology and his motherland.

We declare that we have no conflict of interest.
